# Female cyclists' experiences of saddle sores and their effect on cycling

**DOI:** 10.3389/fspor.2026.1734202

**Published:** 2026-02-03

**Authors:** Louise Burnie, Phil Burt, Kirsty Lindsay, Neil Heron, Paul Ansdell, Elisa Pastorio, Kirsty M. Hicks, Natalie Brown

**Affiliations:** 1 School of Sport, Exercise and Rehabilitation, Faculty of Health & Wellbeing, Northumbria University, Newcastle upon Tyne, United Kingdom; 2 Phil Burt Innovations Ltd., Manchester Institute of Health and Performance, Manchester, United Kingdom; 3 Centre for Public Health, Queen’s University Belfast, Belfast, United Kingdom; 4 Applied, Sports, Technology, Exercise and Medicine (A-STEM) Research Centre, Swansea University, Swansea, United Kingdom; 5 School of Sport and Exercise Science, Welsh Institute of Performance Science, Swansea, United Kingdom

**Keywords:** female health, perinodular indurations, sports injuries, vulva swelling, women

## Abstract

**Objectives:**

The aims of this study are to: (1) understand the type, severity, and experiences of saddle sores in female cyclists, (2) explore the impact on enjoyment, training, and performance, and (3) what prevention or treatment methods female cyclists use, including discussing these issues with their coach, bike fitters, and medical staff.

**Methods:**

20 competitive female cyclists (age 35.1 ± 7.7 years, cycling for 11.9 ± 7.4 years, three elite, seven subelite, and 10 club cyclists) were interviewed using an open-ended, semistructured approach. A thematic analysis was conducted.

**Results:**

Saddle sores were highly prevalent in female cyclists and most occurred in the vulva region. The saddle sores were suggested to be attributed to pressure, friction at saddle contact points, and sweat or a combination of all three. The biggest reported impact of saddle sores was that they reduced the enjoyment of cycling. Participants identified risk factors for developing saddle sores and methods of how they attempted to prevent them from occurring. To treat and manage saddle sores, the participants used treatment creams, modified training, and in severe cases, they sought medical treatment or took a break from cycling. The participants reported the process of finding a comfortable saddle and bicycle setup as trial and error, which was long and expensive. Generally, saddle sores are considered a taboo topic, and many participants in this study were reported to have received poor or conflicting advice on this topic.

**Conclusion:**

These findings highlight the need for improved education on the prevention and treatment of saddle sores and more research into female-specific bicycle setup and saddle design.

## 1 Introduction

Saddle sores are a common cycling injury (1–5). The term saddle sores cover a range of conditions from acute pain, chafing, and bruising to the sit bones, upper thigh, perineum, and vulva (3, 6–9), genital numbness (6), folliculitis and ulceration of the skin (8, 9), chronic unilateral labia swelling/hypertrophy (10–12), vulval nodules or swelling (13), and urogenital overuse injuries and sexual dysfunction (14, 15). Saddle sores can occur in any area in contact with the saddle and develop following microtrauma to the skin caused by pressure and friction during cycling (3, 16). Saddle sores can reduce cyclists' enjoyment of cycling and decrease the frequency and duration of cycling (4, 16).

The cycling community has described saddle sores as a frequent occurrence for female cyclists (3, 5) with 39%–89% of female cyclists reporting saddle sores (6, 14, 17, 18), and it has been suggested that there is a greater prevalence in females (3). There has been an increase in females presenting to gynecology clinics in the United Kingdom with cycling-related vulval symptoms (5). In severe cases, female cyclists might require surgery to treat persistent unilateral vulval swelling (10, 12). Additionally, 52% of female cyclists who experience numbness and/or pain have higher odds of reporting sexual dysfunction (19). Forty-four percent of recreational female cyclists have reported being discouraged from cycling due to vulval/perineal discomfort, resulting in them reducing cycling activity (5). A recent scoping review by Napier and Heron (16) recommended a cohort study to assess the prevalence of saddle sores in male and female cyclists and in different cohorts (e.g., commuters, recreational cyclists, and elite-level cyclists). There are specific anatomical differences between males and females which could explain the suggested greater prevalence of saddle sores in females. Specifically, differences in genitalia and pelvis structure exist as females have a larger distance between ischial tuberosities (20, 21) and a rounder pelvic inlet (22). This could be one of the influencing factors on the differences between males and females in pelvic orientation and movement during cycling (20, 21). Females have been shown to exhibit greater pelvic tilt on the drops (holding onto the lower part of the curved handlebars) and greater internal–external rotation and pelvic obliquity with increasing power output (20). When riding on the drops, female cyclists have a significant increase in maximum pressure on the anterior of the saddle which does not occur in male cyclists (21). It has been demonstrated that when the handlebars are positioned lower than the saddle, which is a typical setup for a competitive female cyclist to limit aerodynamic drag, there is an increase in perineum saddle pressures, a decrease in anterior vaginal and left labial genital sensation (17), and increased genital numbness (19). These differences in saddle pressure distribution between males and females could be a contributing factor to the greater incidence of female cyclists suffering saddle sores. Another contributing factor to the greater prevalence of saddle sores in females is increased labial sensitivity during menses, thereby increasing the likelihood of saddle discomfort and pain when cycling (23).

While research is beginning to emerge in female cyclists to identify the prevalence and cause of saddle sores, the lived experiences are yet to be fully elucidated, including the impact on training and performance. Therefore, this study aimed to (1) understand the type, severity, and experiences of saddle sores in female cyclists; (2) explore the impact on enjoyment, training, and performance; and (3) determine what prevention or treatment methods female cyclists use, including discussing these issues with their coach, bike fitters, and medical staff.

## 2 Methods

### 2.1 Participants

Twenty competitive UK-based female cyclists were recruited by purposive (criterion-based) sampling (24) based on the following: (a) competitive female cyclist, (b) 18 years and older, and (c) assigned biologically female at birth. Participation level was categorized according to McKay et al.’s (25) tier system. The participants consisted of three elite cyclists (tier 4, competed for a Union Cycliste Internationale (UCI)-registered cycling team), seven sub-elite cyclists (tier 3, competed in national-level domestic competitions), and ten club cyclists (tier 2, competed for an amateur cycling club). The participants were aged 35.1 ± 7.7 years (range 21–45 years). Participants’ training history, characteristics, and racing frequency are detailed in Table 1. The participants competed across a range of cycling disciplines (number of participants competing in discipline in brackets) including road racing (11), criteriums (4), time trials (12), hill climbs (3), indoor racing (e-sports) (4), track (1), gravel (1), mountain bike (3) and cyclo-cross (5), with some participants also competing in triathlon (4) and duathlon (2). All participants regularly rode a road bike in training. Eleven of the participants had a coach, of whom eight were male and three were female.

**Table 1:** Participant training and racing characteristics.

Variable	Tier 2—club (10)	Tier 3—sub-elite (7)	Tier 4—elite (3)
Age (years)	40 ± 5	28 ± 6	33 ± 7
Training background (years cycling)	9 ± 6	12 ± 4	22 ± 11
Training frequency (number of sessions per week)	7 ± 3	7 ± 1	8 ± 1
Training volume (hours per week)	9 ± 4	12 ± 4	19 ± 1
Race volume (number of races per season)	9 ± 7	22 ± 12	31 ± 6
Coach [number of participants (%)]	3 (30%)	5 (71%)	3 (100%)

### 2.2 Procedure

The study was approved by the Northumbria University Faculty of Health and Life Sciences Research Ethics Committee (project number: 6056). The participants were recruited through the lead researchers’ networks with national cycling federations, cycling teams, and clubs. They were provided with the details of the study, and written informed consent was obtained. The participants were sent a short pre-interview questionnaire which took approximately 10 min to complete to obtain their demographic information and bicycle and saddle makes and models.

To address the research aim, a combination of epistemological constructionism and ontological relativism to inform an interpretivism research paradigm was adopted (26). The lead researcher (a female cyclist, to help build rapport with the participants) conducted semi-structured interviews in English with open-ended questions to allow participants to express thoughts and expand on topics (26). The first part of the interview asked participants about their menstrual cycle and its effect on training and competition based on questions from Brown et al. (27), which are reported separately. This was followed by questions on bicycle setup, overuse injuries, and saddle sores reported in this current study (see Supplementary Material). The interviews lasted 26.1 ± 7.0 min (range 15.3–44.1 min) and were conducted by the lead researcher either face-to-face or online via Microsoft Teams. Interviews were recorded using Microsoft Teams which also produced a transcript of the interview.

### 2.3 Data analysis

The interview transcripts produced by Microsoft Teams were checked by the lead researcher for accuracy and removal of personal identifying information, and where necessary, small grammatical changes were made to improve the flow of the text. They were then re-read by the lead researcher to ensure immersion in the data. The lead researcher (LB) undertook an initial analysis and coding of the transcripts using inductive reasoning in the software program NVivo (QSR NVivo 14). A thematic analysis was conducted following the steps outlined in the study of Sparkes and Smith (26) which is based on a previous study (28). Phase 1: immersion—the lead researcher (LB) immersed herself in the data by rereading the interview transcripts. Phase 2: generating the initial codes—the lead researcher systematically went through the interview transcripts and inductively coded the data into initial codes. Phase 3: searching for and identifying themes—the lead researcher then grouped the initial codes into main and sub-themes. Phase 4: reviewing themes—the lead researcher then reviewed, reworked, and refined the themes by repeatedly reviewing the generated themes and the original data to check that the themes generated captured the essence of the underlying data. Phase 5: defining and naming themes—the lead researcher then reviewed the theme names to check they accurately and concisely summarized the essence of each theme. Phase 6: writing the report—the lead researcher wrote the combined results and discussion sections using this as a further opportunity to refine the themes. A second researcher (NB) acted as a critical friend (29), by reviewing interview transcripts and initial codes and themes. NB and LB then discussed the themes resulting in a few changes to the themes and theme names before the final themes were agreed. The whole research team reviewed the themes, results, and discussion section and agreed with the final themes as decided by LB and NB.

## 3 Results and discussion

Through thematic analysis of the interview transcripts, 7 main themes emerged and 38 sub-themes: prevalence, type, location, severity, and impact of saddle sores; risk factors for saddle sores; saddle sore treatment and management; prevention of saddle sores; saddle choice and fitting; bicycle setup; and comfort in discussing saddle sores (Table 2). Each of these themes is discussed below.

**Table 2:** Themes.

Main themes	Sub-themes	Illustrative quotes
Prevalence, type, location, severity, and impact of saddle sores	Prevalence	“Terrible saddle sores. All the time.” (P17, club)
	Severity	“A bit tender, just normal swelling, but it's nothing like what I've experienced previously. It's manageable.” (P5, club)
	Type	“Mine are like little spots, and then they get bigger and go into boils and then they get infected, so mine are quite severe ones.” (P14, elite)
	Location	“Mine are more like the crease between my thighs and my bits. Before changing my saddle, I felt like it squashed the inside.” (P17, club)
	Impact	“I just crack on with it. I mean it definitely affects enjoyment.” (P12, club)
Risk factors for saddle sores	Cycling volume	“Definitely the length of time, if I come back from a 7/8 h ride, I'm much more likely to have done something than if I just go out for a couple of hours.” (P12, club)
	Ill-fitting shorts	“I opt for a boys’ pad now because the ladies’ pads are too padded, and it just works its way into places I don't want it to be and causes friction, and it ends up really sore.” (P5, club)
	Ill-fitting saddle	“It's the left-hand side [swollen outer labia], and that really stems from being made to ride saddles that didn't fit me or didn't work.” (P18, elite)
	Time trial position	“Sometimes I do [get saddle sores], especially with time trialling where you can't really shift about on the saddle that much.” (P8, sub-elite)
	Indoor static cycling	“Because you don't have the same level of movement, the bike is more fixed, so it's harder to shift around… collectively the team starts to complain that it's uncomfortable.” (P15, club)
	Rough road surface	“Rough tarmac is uncomfortable to rattle over.” (P9, club)
	Wet weather conditions	“We had a really wet stage race, some girls actually dropped out of the race [due to saddle sores], and that made it [saddle sores] much worse and then after that I had to have some days off.” (P10, elite)
	Asymmetries	“We worked out that I have one leg shorter than the other, I always had saddle sores on one side.” (P8, sub-elite)
Saddle sore treatment and management methods	Treatment creams	“I would use some light [chamois] cream on the shorts with some Sudocrem on the sores day-to-day.” (P19, club)
	Medical treatment	“I’ve had a few drained actually. I’ve been on various antibiotics.” (P14, elite)
	Break from cycling	“I couldn't even train on the bike I was having to swim and walk, just for it to settle down.” (P1, sub-elite)
	Modify training	“I’ve done lots of out-the-saddle turbo sessions. Lots of running’ (P14, elite
	Change saddle	“Another bike fit and a different saddle.” (P1, sub-elite)
Prevention of saddle sores	Well-fitting cycling shorts	“The shorts are beautiful, it is a women-specific pad. They are so comfortable.” (P6, sub-elite)
	Anti-chafing cream	“We got this thing here [shows chamois cream] and it's really good.” (P13, club)
	Hygiene	“Lots of sweat, that's usually bad [for developing saddle sores]. I rarely go for coffee rides because I don't like to sit in my shorts.” (P14, elite)
	Not shaving pubic hair	“I know if I shave, I will get lumps. I’ll get spots, so I use beard trimmers.” (P18, elite)
	Changing position while riding	“I try and get up and down, an extra thing to help.” (P2, club)
	Well-fitting saddle	“I have not had one issue since I changed saddle, and I’ve made the effort to really invest in a good bike fitter.” (P18, elite)
	Lack of information	“I’m quite interested to know what to do to prevent saddle sores.” (P12, club)
Saddles choice and fitting	Saddle shape	“I’ve got the narrowest ISM [saddle] that you can get, but I still will [cable] tie the front nose.” (P10, elite)
	Role of bike fitting	“I had a bike fit and he said I needed a different saddle because he was looking at the pressure for the saddle I was on, but he wasn't very helpful at all in terms of knowing actually what to do. He didn't have any women's specific advice.” (P13, club)
	Recommendations	“I wasn't getting on with my saddle and I tried my friends, and it was really good.” (P4, sub-elite)
	Online research	“I did a lot of Googling and then tried a few different saddles that were recommended on there.” (P12, club)
	Saddle sponsors	“They’re really looking to find the best options for the team because we had really big problems in the first year of the team with saddle sores.” (P10, elite)
	Trial and error	“I have asked around before, getting recommendations from other female cyclists and seeing what they’re using and just buying some second-hand saddles, trying them, if they're no good, sell them on again.” (P1, sub-elite)
	Cost	“If there was another way to find your perfect saddle without spending a fortune I would generally be interested.” (P19, club)
Bicycle setup	Bicycle types	
	Bicycle fitting	“I always have a tilt [downwards]. It's 2 degrees, that's essential if I don't have a tilt, then it's just uncomfortable.” (P14, elite)
	Role in saddle comfort	“The bit underneath my seat is modified because I’m more comfortable further forward.” (P17, club)
Comfort in discussing saddle sores	Coach	“They didn't care too much, so I tried to solve it myself, because maybe they think it's OK, it's normal.” (P7, sub-elite)
	Male bike fitters	“So, there aren't many female bike fitters right? So, I feel that when young riders, especially female riders, go to a bike fitter, they won't speak up.” (P18-elite)
	Uncomfortable discussing	“[I didn't discuss saddles sores with my coach], which with hindsight obviously was a mistake.” (P9, club)

### 3.1 Prevalence, type, location, severity, and impact of saddle sores

All of the participants reported having suffered saddle sores, which is higher than previously reported where 39%–89% of female cyclists reported saddle sores (6, 14, 17, 18, 30). Previous research has typically involved larger cohorts than this study and was often part of an assessment of all overuse injuries in cycling. Therefore, we acknowledge that one part of this study was focused on researching saddle sores; this may have contributed to a recruitment bias, where cyclists who suffered from saddle sores were more likely to take part. The level of cyclists included in the current study is also a key consideration, as higher-level cyclists spend proportionately more time on their bike training (31), increasing the risk and likelihood of experiencing saddle sores which may explain the variation compared with previous literature.

I get saddle sores even though I really like my saddles. I don't really know why, but I often get saddle sores. (P4, sub-elite)

Terrible saddle sores. All the time. (P17, club)

Nine of the participants who get saddle sores have said their saddle sores are manageable, but this involves them tolerating some pain, discomfort, swelling, and chafing:

I do get them [saddle sores] particularly when doing lots of hours, but it's kind of that slight middle ground where it's never really that bad to bother doing anything about it… In training I'll do endurance, and they’ll be getting worse over the days, you get on the bike and you’re that's a bit uncomfortable, but you get going and it's fine, but then you have a rest day and… It actually recovers quite a lot. And then by the time you get back on your bike the next time it's kind of fine again. So, generally it's not too much of a problem. (P10, elite)

The one [saddle] that I'm on now, it doesn't break the skin, it doesn't damage anything internally; it's just a bit of tenderness on the pressure points. And obviously, if I've been on the bike for a long time, especially on the turbo [indoor training on a static bicycle], and I haven't moved around much, then I get it's almost like a hardish lump that's a bit tender, just normal swelling, but it's nothing like what I've experienced previously. It's manageable. (P5, club)

Three participants have resolved their saddle sore problems and do not currently suffer from saddle sores:

I never really get saddle sores… I have had saddle sores in the past but with this saddle I have now, I've never noticed it. (P11, sub-elite)

The type of saddle sores suffered by the participants was wide ranging, and causes were suggested to be due to pressure, friction at saddle contact points, and sweat or a combination of all three, which is similar to the causes suggested by medical consultants (3). The types of saddle sores and vulva symptoms reported by participants were similar to those reported previously by female recreational cyclists (5). Sixty percent of the participants reported suffering saddle sores that were spots, boils, cysts, lumps, and ingrown hairs, which for some participants then became infected:

Mine are like little spots, and then they get bigger and go into boils and then they get infected, so mine are quite severe ones. (P14, elite)

I had an infection, and I think essentially it was an ingrown hair that then got massive and infected, and I just felt really ill, and I was put on antibiotics. (P20, club)

Fifty-five percent of participants reported suffering discomfort, tenderness, numbness, pain, and swelling in the areas in contact with the saddle such as the vulva, upper thigh, and sit bones.

I’ve done races where I've just sat in a tuck position, and I've gone completely numb down there. (P18, elite)

You can sometimes feel after a session with a lot of efforts where you've really dug deep, a little bit of soreness or tenderness. (P16, sub-elite)

I do get a sort of external pressure point bruising or swelling if I'm doing a particularly long session, so the day after getting back on the bike again… getting back into that position, waiting for the lips to go numb. (P5, club)

Some participants also reported suffering blisters and chafing:

Sometimes the ones on the outer labia can be blistery. I had one relatively recently, I could tell the skin was really thin, so I popped it with a needle. I have occasionally had a slight blister on the inner labia. (P8, sub-elite)

Three participants had suffered permanent unilateral vulva hypertrophy where the outer labia is larger and swollen on one side because of cycling. This has been reported previously as a medical issue that affects female cyclists (10, 11). Vulva swelling is speculated to be caused by repeated microtrauma from vibrations and locations of high pressure due to poorly fitted saddles and biomechanical imbalances and asymmetries resulting in loading one side of the vulva more than the other (10–13). The participants suggested that their unilateral vulva hypertrophy was the result of riding poor-fitting saddles for their anatomy and riding style, riding in a time trial position, and prolonged indoor static training. Two participants had sought medical advice, but they reported receiving poor or conflicting advice.

Outer labia just on one side, so I don't know why it was on one side if I was like riding at a funny angle or if as a man unsolicited at a race said, a lot of women have a labia imbalance. I think with hindsight it's when I first started turboing. When I started doing a lot, it came on moderately quickly. So, I went to the doctors and she said women don't get saddle issues, but men do. I said my concern is it doing lasting damage? And she said no. Turns out she was actually wrong, and it has done lasting damage, but she was a doctor, so at the time I believed her… I've been to see a gynaecologist about it, they said although they could do surgery, they thought it could potentially be worse with the scar than it would be from having the swollen tissue, so I've just left it as it is, and I guess I'm not cycling as much anymore. (P9, club)

The participants reported saddle sores most occurred in the vulva region (65%) predominantly the outer labia, but some on the inner labia and urethra. This is similar to previous reports in female cyclists where the labia and vulva were the most common location for saddle sores in female cyclists (53%) (6).

I was trying some different ones [saddles] and the nose of the saddle basically pushed the chammie [chamois pad—padding within cycling shorts] up like inside and then I got like a blister, literally where I wee and that was painful. So, I quickly took that saddle off. (P10, elite)

Forty percent of the participants reported saddle sores at the upper thigh, which is higher than previously reported (22%) (6); however, this should be interpreted with caution due to variation in methodologies (interviews vs. surveys):

Mine are more like the crease between my thighs and my bits, so right in there… Before changing my saddle, I felt like it squashed the inside. (P17, club)

Thirty-four percent of the participants reported saddle sores on the buttocks mainly the sit bones but also the gluteal fold, again higher than previously reported (22%) (6):

The one that I had going on for ages was on my sit bone, right in the middle, so that wasn't in a great place. (P14, elite)

The seam from the edge of the chammie. So, the bum crease, that's where I tend to get them. (P10, elite)

The higher percentage of participants reporting saddle sores in different locations is probably because all participants in this study reported suffering from saddle sores in comparison with Gaither et al. (6), where only 39% of the female cyclists said they had suffered saddle sores. This may be due to the different methods in gathering the data. Gaither et al. (6) used a survey which does not allow the researcher to probe the participants for detail or clarify with the participants who may have a different understanding of what constitutes a saddle sore, whereas interviews allow a much richer capture of participant experiences (32).

The biggest impact of saddle sores was that they reduced the enjoyment of cycling due to pain and discomfort (4), with 75% of the participants reporting they just put up with the discomfort and pain of saddle sores.

Maybe you want to finish your training because of the saddle not because of your fatigue. So, it's more uncomfortable than affecting performance. (P7, sub-elite).

I just crack on with it. I mean it definitely affects the enjoyment. It's much more enjoyable if it's completely pain-free, but I tend to just get on with it unless it's really bad. (P12, club)

In more severe cases, the participants reported having to take a break from cycling due to saddle sores which can result in loss of fitness (5, 16).

### 3.2 Risk factors for saddle sores

Long bicycle rides were the most reported factor to increase the risk of saddle sores developing along with high training volume agreeing with previous research (3, 5, 12, 16). Elite cyclists typically have higher weekly cycling volumes compared with club cyclists (Table 1), potentially putting them at higher risk of saddle sores.

Definitely the length of time, if I come back from a 7/8 h ride, I'm much more likely to have done something than if I just go out for a couple of hours. (P12, club)

Another risk factor mentioned by the participants was ill-fitting cycling shorts and poor chamois design:

I opt for a boys’ pad now because the ladies’ pads are too padded, and it just works its way into places I don't want it to be and causes friction, and it ends up really sore even with the balm on the chammie. (P5, club)

Another risk factor reported was ill-fitting saddles for a rider’s anatomy and riding position and poor saddle design which has been previously reported (16).

It's the left-hand side [swollen outer labia], and that really stems from being made to ride saddles that didn't fit me or didn't work. So, for example, I wasn't allowed to change saddles, and there weren't that many saddles on the market for women back when I was racing as a junior. (P18, elite)

One participant commented that it is often the combination of the cycling shorts and saddle that increases the risk of saddle sores developing:

I think that it's not necessarily just the saddle that's a problem or just your bib shorts. It's usually a combination of the two together where the seam sits in relation to the saddle. (P10, elite)

The participants reported that time trial position and riding on the drops increased the risk of saddle sores particularly in the vulva and upper thigh region:

Sometimes I do [get saddle sores], especially with time trialling where you can't really shift about on the saddle that much. I have had a few races where I have been like hovering for half of it because you just can't put weight on that part [vulva] which hasn't been great. (P8, sub-elite)

This agrees with previous research that has found having the handlebars lower relative to the saddle increases perineum saddle pressures and decreases genital sensation (17, 19). Additionally, when riding on the drops, female cyclists have a significant increase in maximum pressure on the anterior of the saddle which does not occur in male cyclists (21).

The participants also reported indoor cycling on a static trainer as a risk factor for saddle sores and increasing discomfort due to the fact that you do not move around and change position as you would on an outdoor ride:

I say we—the team [female indoor cycling team] definitely complains. So, 25–30 km [indoor e-] races, which are about an hour are doable. Anything over that starts to get uncomfortable. Just because you don't have the same level of movement, the bike is more fixed, so it's harder to shift around. You don't have a traffic light or a reason to stop… So you're sat. So, anything over about 30 km, collectively the team starts to complain that it's uncomfortable. (P15, club)

Another factor that participants mentioned influences saddle discomfort and pain is rough road surface and off-road cycling which has previously been reported to increase the odds of saddle sores developing compared with urban road cycling (6).

When I'm approaching any kind of a rough road surface I get out the saddle. Rough tarmac is uncomfortable to rattle over. (P9, club)

Wet weather was another factor mentioned by a few participants that increased the risk of saddle sores developing:

We had a really wet stage race, some girls actually dropped out of the race [due to saddle sores], and that made it [saddle sores] much worse and then after that I had to have some days off. (P10, elite)

Another risk factor mentioned by a few participants was that biomechanical or strength asymmetries can increase the risk of one-sided saddle sores, and this has been highlighted previously (4).

After a while we worked out that I have one leg shorter than the other, I always had saddle sores on one side. Now I've got a shim on my pedal. I get them on both sides, but less regularly. (P8, sub-elite)

### 3.3 Saddle sore treatment and management methods

A popular treatment for saddle sores used by 65% of the participants was treatment creams in particular Sudocrem (barrier and antiseptic healing cream for skin), which has previously been mentioned by UK female recreational cyclists (5):

A recent [cycling] trip, cycling 6 days in a row. I would use some light [chamois] cream on the shorts with some Sudocrem on the sores day-to-day. (P19, club)

For more serious saddle sores, six participants had sought medical treatment such as medicated creams with steroid formulations or antibiotics for infected saddle sores (3, 16). As mentioned previously, saddle sores are not well understood by general medical practitioners or gynecologists, and four participants reported receiving poor or conflicting advice:

I did go to the doctor's once because I had one that was repeatedly coming back but to be honest, they were not very useful. It's really difficult when you're an athlete going to the doctors about something because the response is just don't ride which at that period I think I'd had a month off the bike, and it was still there. (P4, sub-elite)

One participant's large saddle sores required surgical drainage several times. A combination of excess fluid drainage and antibiotics is standard treatment for this type of sore (16).

I've had a few drained actually. I've been on various antibiotics, but now I'm on a longer-term antibiotic. I have been on it for two years but then now they've changed it to a different one because they said I might have become resistant to it, so it's a very low dose but it just keeps my skin at bay. (P14, elite)

In line with Bury et al. (3), the participants reported that for severe saddle sores, they took a break from riding their bicycles or missed some sessions. However, they tended only to do this for severe cases to avoid missing training and the potential loss of fitness (16). This has the potential to have a greater impact on elite cyclists as cycle racing is their occupation; taking a break from cycling could mean missing races and also loss of fitness which could impact race results on return from saddle sore injury and professional cycling contract renewal.

Usually when I have a saddle sore it's because I'm run down and it's because I'm tired or I'm stressed, so I've found if I do take time off, it does help it go away quicker because then I get less run down. (P14, elite)

So, before racing a National Series Road Race I had to have my saddle nose really tilted down, training wise I couldn't even train on the bike I was having to swim and walk, just for it to settle down… I had a week off. (P1, sub-elite)

Additionally, the participants reported modifying their training either by reducing cycling volume by making sessions shorter, riding out of the saddle, or cross-training off the bicycle. Again, modifying training has previously been reported as a method to manage saddle sores (16):

I've done lots of out-the-saddle turbo sessions. Lots of running…. He [my coach] changed all my training. (P14, elite)

There have been times in the past where I felt perhaps a bit sore, so we've had to slightly modify a session or maybe I do an off-bike warm-up to reduce the time on the bike, just to stop it [saddle sore] flaring up more. (P16, sub-elite)

One participant reported cold water helped for bruising and swelling-type saddle sores, and it has been recommended to apply a cold compress to the vulvoperineal region to reduce swelling (12).

Another participant who suffered one-sided saddle sores found that addressing leg length difference with a pedal/cleat shim and physiotherapy to address strength imbalances resolved their one-sided saddle sores. Addressing asymmetries was one of the key recommendations of Fallon et al. (4) to help prevent saddle sores.

Unsurprisingly, when participants experienced saddle sores, they would often change their saddle, sometimes going for a bike fit to address this issue. This is in line with previous recommendations that cyclists have a professional bike fit to adjust position to help address saddle sore issues (4, 5).

Another bike fit and a different saddle. I've changed saddles again since, so both of those things really. (P1, sub-elite)

Most participants treated and managed saddle sores by themselves, sometimes talking to their coach to get training modified or a bike fitter to help advise with saddle choice and bicycle setup. Several researchers have commented on the lack of quality information and education for cyclists, coaches, and medical practitioners on treatment, management, and prevention of saddle sores for female cyclists (3, 5, 16, 18). Harrison and Edey (5) highlighted that effective management of early symptoms may prevent more significant symptoms from developing that potentially require surgical management or cause permanent damage.

### 3.4 Prevention of saddle sores

The participants highlighted that well-fitting cycling shorts with a chamois pad that suited their anatomy helped prevent saddle sores. This has been reported previously by other cyclists as helpful (5) and has been recommended by medical consultants to help prevent saddle sores (3, 4, 10, 16).

There's some of the cheaper shorts with a really light thin pad [chamois] or the pad moves. I hate those. But the kit that we've got for the team, the shorts are beautiful, it is a women-specific pad. They are so comfortable, which is quite fortunate. (P6, sub-elite)

One participant highlighted that not wearing underwear beneath cycling shorts helped improve saddle comfort. It has been recommended that cyclists do not wear underwear beneath cycling shorts (4, 16). However, as this participant highlighted, this is not known to novice cyclists, and therefore education is required.

At some point, I did research into whether people wore knickers or didn't, so I asked all the women who cycled and 50% did and 50% didn't. So eventually I managed to accept that it was OK to not wear knickers, so that helped. (P13 club)

Forty percent of the participants reported using anti-chafing or chamois creams to help prevent saddle sores which is similar to previous reports by female recreational cyclists where 37% used chamois cream (5).

We got this thing here [shows chamois cream] and it's really good. Now I would use it if ever I'm going out for a long ride. (P13, club)

Medical consultants have recommended using anti-chafing creams (3), and a review by Napier and Heron (16) found the most commonly cited method to prevent saddle sores was using chamois cream. However, there is a lack of evidence for the use of anti-chafing creams as the only study that assessed anti-chafing creams did not have a comparison control group where the cyclists used no creams (7). Additionally, there have been reported negative effects for some riders with chamois creams causing skin pore blockage leading to folliculitis (16), as reported by one participant in this study:

So, I found I was using more and more [chamois cream] which I think had a reverse effect which is block the pores—caused more chaos. I stopped using it at all. (P19, club)

Several participants mentioned personal hygiene and getting showered and changed straight after completing a ride, and avoiding café stops was important to prevent saddle sores from getting infected. Good personal hygiene has been recommended by researchers and medical consultants previously, e.g., showering straight after cycling and removing and washing the kit to prevent infections associated with saddle sores such as folliculitis and ulcerations (3, 4, 16). There is a lack of objective evidence to support this recommendation; however, several participants reported that this was important:

Lots of sweat, that's usually bad as well [for developing saddle sores]. I rarely go for coffee rides because I don't like to sit in my shorts. (P14, elite)

Several participants mentioned how stopping shaving pubic hair had a massive beneficial effect if they suffered spots and ingrown hair type saddle sores, and this has been recommended by medical consultants and other female cyclists (3, 5).

I know if I shave, I will get lumps. I'll get spots, so I use beard trimmers. So, I'm aware of what I need to do so I've not had in a very long time any spots. (P18, elite)

I stopped shaving, and actually that seems to have cleared it up, before I just kept on getting saddle sores and it was just horrendous… So yeah, game changer. (P6, sub-elite)

It has been suggested that elevating the lower limbs during rest periods to promote lymph drainage of the perineum and pelvis and physiotherapy to simulate alternative lymph drainage pathways could help prevent vulva lymphedema (12). However, none of the participants in this study mentioned either of these prevention and management methods.

Several participants suggested changing riding position and pedaling out of the saddle for periods of a ride can help prevent saddle discomfort. Previous research has found that standing >20% of the time when cycling reduced the odds of genital numbness (6). Additionally, as mentioned previously, addressing strength and mobility asymmetries in the lower body and leg length difference can prevent saddle sores (4).

One of the key prevention methods to stop saddle sores from developing is getting a saddle and bicycle setup that suits a rider's anatomy and riding position (3, 16), as highlighted by this participant:

In the last four years, I have not had one issue since I changed saddle, and I've made the effort to really invest in a good bike fitter. (P18, elite)

It has been suggested that females use a wider saddle without an anterior nose (10). However, four participants reported suffering saddle sores specifically when they rode a wide saddle without an anterior nose. Often, these saddles are too wide for a rider's anatomy, as reported by this participant who cable-tied the nose of the saddle to make it narrower:

I've got the narrowest ISM [saddle] that you can get, but I still will [cable] tie the front nose, which is not recommended, but it makes it better for me. (P10, elite)

Several participants talked about the lack of information on female saddle sores and the difficulty in finding advice on how best to prevent and manage saddle sores and guidance on saddle choice for female riders.

I guess I'm quite interested to know what to do to prevent saddle sores. I would be interested to read advice on that. (P12, club)

I don't think there's enough [research] on female-specific saddles and positions because I know when I wanted to change a saddle recently there was a couple of articles and videos online, but there was nothing with clear research which showed what saddle style is best for women. (P16, sub-elite)

Fifty-five percent of the participants in this study highlighted that they want more research into female saddle design and bicycle setup to prevent saddle sores and make it easier to find a comfortable saddle. This echoes the call for more research and products designed to address female saddle sores by Great Britain female cyclists (33). In the scientific literature, saddle sores are a male-dominated issue (16), and there is limited quality research into female-specific saddles (5). The existing research has various limitations: small sample size, limited time of participants riding the saddles [can be as little as 4 min, only two studies had a follow up after real world riding (8, 34)], some studies were observational so participants were riding their existing saddle, lack of standardized bicycle setup, and lack of variables measured [in particular, pelvic and vulva morphology (21, 35) and effect of pressure on vulva tissue oxygenation and vibratory thresholds (34)] to help determine what makes a saddle more comfortable (8, 17, 34, 36–39). This lack of underpinning research results in a shortage of information and guidance for female cyclists.

What I would be interested to read is actual proper research on how women sit on bikes. I'd love to see the results for a whole host of different shapes of women with a whole range of different saddles and see what sort of shape [saddle] roughly each woman rode, what they felt like, that would be interesting. (P10, elite)

So, I think how women sit on a saddle, the different shapes of people because people are so different… I really feel that there needs to be more research in the effects on women when they sit on everything because I've done races where I've just sat in a tuck position and I've gone completely numb down there. I've not seen any research on how women sit on saddles. (P18, elite)

Speaking to friends this is something that is really common in women's cycling. I've a friend who's a pro cyclist and she sometimes has had to have periods of time off, because of saddle sores. And she's gone to XYZ doctors and everything. And this is her job. It stops her from being able to compete, so I think it's, it is definitely something that would be fantastic to have more knowledge in. (P4, sub-elite)

I think it just feels like there needs to be a bit more work on saddles for women… Groups of women, they all suffer from a range of issues. So, there's clearly more work needs to be done there. (P19, club)

### 3.5 Saddle choice and fitting

The participants rode 2 ± 1 different types of saddles across their bicycles. Five of the participants rode saddles sold as women's specific only, three participants rode a mixture of women's specific and unisex (or men's) saddles, and twelve participants only rode unisex (or men's) saddles. This is different from what has recently been reported for national-level Spanish cyclists where there was a 50% split between women's specific and unisex; however, more experienced cyclists tended to favor a unisex saddle (30). The saddles were from 14 brands and varied in saddle shape, but 95% of the participants rode saddles with a central cutout or channel through the middle of the saddle, with some saddles designed specifically for riding in time trial position or aggressive aerodynamic road position. This is a large change from what has previously been reported, where only 45% of female cyclists used a saddle with a cutout and the rest a traditional saddle which is solid and domed along its length (37). This reflects the development in saddle design and the greater range of saddles available over the last 15 years.

Different shaped saddles have been demonstrated to influence peak and mean saddle pressures and the location of peak pressures (37–39), genital–perineal vascular perfusion (34), and also saddle comfort (8, 30). Saddles with cutouts or channels can relieve pressure on the center of the vulva and soft tissues which is important as the vulva is not designed to be weight-bearing (5). However, previous research has demonstrated that a cutout saddle can increase mean perineal saddle pressures with the highest pressures along the edge of the cutout (37). In this study, although 95% of the participants rode a saddle with a cutout, 85% still suffered problems with saddle sores. A female-specific saddle or cutout may not resolve the issues. There is a wide variety in vulva morphology (35, 40), so it is highly unlikely that one saddle design will suit all females, and this is probably why saddle type has not been shown to be associated with the prevalence of saddle sores (6).

Eight participants had specifically had a bike fit due to saddle sores and discomfort. The bike fitters used two systems to try and identify suitable saddles. These were systems to measure the sit bone (ischial tuberosity) width and saddle pressure mapping. However, as mentioned above, vulva morphology is also important in saddle fitting and is probably a reason why a participant reported that having a saddle chosen solely on sit bone width was not successful:

I've had one of those things where you sit on … It's got padding in it and it shows you where your sit bones sit. And that's why I got the wide one [saddle]. So, I was on that for a while, kept getting saddle sores. (P8, sub-elite)

Saddle pressure mapping can assist a bike fitter in identifying locations of high pressure that may indicate the likelihood of a saddle sore developing (4), which can help in saddle selection and bicycle setup:

I have had problems before, and we did some pressure mapping and then tried to adjust the position of how I sit. (P10, elite)

However, although saddle pressure mapping can be helpful, as this participant mentioned even after trying lots of saddle options, the best option still didn't provide a perfect solution, so the success of the bike fit also depends on having appropriate saddle choices for females and highlights the need for more research into female saddle design:

I even went to one of those expensive ones [bike fits] where they put you on a sort of electronic pad, so they can see where the pressure is. And even like after trying 30 saddles, the one that looked the best, the best he could find, you could still see in the picture, it wasn't perfect, so I thought I'll try it and maybe see, you know that was just fine tuning, but it was never perfect. (P19, club)

Several participants reported receiving poor advice from the bike fitter:

I had a bike fit and he said I needed a different saddle because he was looking at the pressure for the saddle I was on, but he wasn't very helpful at all in terms of knowing actually what to do. He didn't have any women's specific advice. So, I came away a bit frustrated about that. (P13, club)

Often participants were frustrated that they had not been able to find a comfortable saddle and therefore rode a saddle that for them the discomfort was manageable:

So, I hate everything to do with saddles because I never know if the new ones going to be better. So, at the minute on my summer road bike, I've been thinking about changing it for quite a long time, but then I think is it bad enough to change or now that I am not cycling as much, is it worth risking something that's worse. I think ultimately, I probably should have one comfortable saddle… I'm never convinced that I've got exactly the perfect saddle. (P9, club)

The participants often asked for saddle recommendations from other riders:

The one that I've actually settled on is because I was in Spain with some friends, and I wasn't getting on with my saddle and I tried my friends, and it was really good, and I bought it off them so now I'm hooked, and I buy them on eBay when they're cheap. (P4, sub-elite)

Tried a couple of different saddles and then a very good friend of mine in the US who races for my team indoors. I went to visit her and she had this saddle and we went out for a ride and I was that may actually be the most comfortable saddle I've ever sat on, I mean it's always relative I find with race saddles I'd rather sit on the sofa. (P15, club)

Another method used by participants to try and find a comfortable saddle was researching online for information on saddles. However, this can be limited because of no specific advice based on individual anatomy.

I did a lot of Googling and then tried a few different saddles that were recommended on there and just getting on pretty well with the Selle Italia one that I've got now. (P12, club)

Ultimately, it was the pressure at the front that was the problem, so I Googled and got loads of information… I'd been asking all these people, a national bike shop and bike fitter and whatever, and no one mentioned any of this, but it all made sense. I thought the Pro Stealth from all my research was going to be the answer, but it just wasn't, so I guess everyone is different. (P13, club)

Some participants rode for teams with saddle sponsors, and this could create challenges in getting a comfortable saddle, as two riders reported they had to ride a team saddle even though it gave them severe saddle sores:

I went through a whole thing of ordering new saddles and trying them out. And then I managed to find the Specialized one that fits really well. And then I moved teams, and I couldn't use it. (Interviewer: That really surprised me that you were not allowed to change saddle for health reasons.) Yeah, teams can be like that though. (P14, elite)

Whereas another participant reported that, because several members of their team had suffered severe saddle sores on team sponsor saddles, they were allowed to choose their own:

So, saddles have been a really big topic each year with the team. They're really looking to find the best options for the team because we had really big problems in the first year of the team with saddle sores, and we had riders have to drop out of stage racing and stuff because the saddle sores have been so bad. So, whilst we have a saddle sponsor, it's still very free rein, you can try their saddles… They look at your seat bones, look at your position on the bike, look at what saddle could be good, but if you need to have your own saddle then you have your own saddle. (P10, elite)

Only the elite riders reported having a saddle sponsor for their teams; therefore, this is an issue that is only relevant to professional cyclists, and the influence on riders' comfort depends on the professional cycling team's approach to saddle choice.

Ultimately, for most participants (65%), their current saddle was chosen through a process of trial and error often based on bike fits and recommendations. Due to the complexity in determining the best saddle and setup for an individual rider, this is often the recommended process (4).

I have asked around before, getting recommendations from other female cyclists and seeing what they're using and just buying some second-hand saddles, trying them, if they're no good, sell them on again. But yeah, tried different ones, even on a bike fit you get put on a couple of different saddles. I just know straight away. I'm literally like absolutely not. (P1, sub-elite)

However, several participants mentioned that the long trial-and-error process of purchasing and then trying different saddles, going for bike fits, and modifying bicycle setup is expensive and can be a barrier to finding a comfortable saddle and bicycle setup. This in particular can deter recreational cyclists or those from lower-income backgrounds preventing them from cycling if they cannot find an affordable and comfortable setup (5).

It's taken a long time really to find the right equipment for me that fits, but now I'm super comfy. So, it's trial and error. It's having the money to buy different saddles and trying them. (P18, elite)

If there was another way to find your perfect saddle without spending a fortune I would generally be interested. I don't think it's [my saddle] is perfect, I've done all sorts of things in the past. (P19, club)

### 3.6 Bicycle setup

The participants owned on average 3 ± 1 bicycles from a range of 27 manufacturers. Four of the participants rode bicycles sold as women's specific only, six participants rode a mixture of women's specific and unisex (or men's) bicycles, and ten participants only rode unisex (or men's) bicycles. Fourteen of the participants had a professional bike fitting. Typical changes participants had made to their bicycle setup were narrower handlebars, shorter crank length, stem length changes, and changing saddles. Some of the participants struggled to describe specifically what had been changed on their bicycle because their partner or bike fitter had made the changes.

Bicycle position can influence saddle comfort (3), so this needs to be assessed in conjunction with saddle choice. It has been suggested that having the saddle too high may tilt the pelvis laterally to a rider’s dominant side, which could increase the risk of saddle sores on one side (4). A couple of studies have recommended the saddle nose tilted slightly downwards to reduce the incidence and symptoms of saddle sores (4, 10). Of the eleven participants who discussed saddle angle, six had a downward tilt and five a level saddle. One participant mentioned a downward tilt was important for comfort; however, another participant noted that for them a level saddle was better for power production:

I always have a tilt [downwards]. It's 2 degrees, that's essential if I don't have a tilt, then it's just uncomfortable. (P14, elite)

I'm very particular about the angle of my saddle on my TT bike if anyone touches my saddle I sit there with my spirit level, it has to be exact, it's just completely flat. I find that when I tilt the saddle a really small amount up or down it has a massive impact for me where I can deliver power whether it's from above my knees or whether it's coming from my glutes or whatever. (P10, elite)

Several participants mentioned the role of bicycle position in conjunction with the saddle to improve comfort while in an aerodynamic position for performance.

I'm sure the bit underneath my seat is modified because I'm more comfortable further forward and it was to bring my saddle [forward]… because I'm more comfortable, right on the tip of my saddle. (P17, club)

### 3.7 Comfort in discussing saddle sores

Six of the participants mentioned discussing saddle sores with their coach, often so the coach could modify their training and to get alternative saddle recommendations.

So, before I cable-tied the nose of my TT bike, I was getting pretty bad saddle sores on the long endurance as when the weather is really bad, I needed to do endurance on my TT bike inside. So, we just had to make the sessions shorter and then cable-tied the nose. We'd tweak the session to be more comfortable. I have told him before and after stage racing. I'll say I'm really sore. I might need a day off because I need to let my saddle sores get better. (P10, elite)

One participant mentioned her coach was not interested in the fact that she suffered saddle sores:

They didn't care too much, so I tried to solve it myself. Yeah, because maybe they think it's OK, it's normal. (P7, sub-elite)

Several participants said they do not discuss saddle sores with their coach as they do not want to miss training:

[I didn't discuss saddle sores with my coach], which with hindsight obviously was a mistake, but I just thought I need to get faster. (P9, club)

One participant highlighted that most bike fitters are male which can be a barrier to conversations, as female riders may be embarrassed or uncomfortable discussing exactly where is sore. It has been reported that elite athletes do not discuss saddle sores with support staff and saddle sores are deemed a taboo subject (18). Additionally, some male bike fitters can lack understanding of female saddle sores, and several participants reported being given poor advice. This highlights the need for education for coaches and bike fitters to provide female riders with appropriate advice on saddle choice and bicycle setup and guidance on how to broach this topic with female riders and the correct terminology for female anatomy.

So, there aren't many female bike fitters right? So, I feel that when young riders, especially female riders, go to a bike fitter, they won't speak up and they say, well, he said that's right. And I'm like, it depends whether you got an inner or an outie [inner labia]. There are so many factors that men will not understand, and I try and encourage all my riders to be really open and honest. If it's not comfortable, you have to say so. I went through with my bike fitter. He's great. I've seen him twice a year for the last five years. I feel like this on the bike. One side is a little bit. I get sore if I do this or that, and I want to sit like this, if I sit like this, it squashes everything. I'm a really big advocate of men learning and understanding as well, but the sport is changing for the good and I feel that there are a couple of women fitters out and about. (P18, elite)

### 3.8 Limitations

It is important to note some limitations in the application of the current findings. Although a relatively small sample size, this is a unique population group, and given the high level of athletes included, it limits the sample size available to interview. All the participants in this study were UK-based, and therefore their experiences may not be representative of cyclists in other countries and from different cultural backgrounds. This study does not include older female cyclists who are perimenopausal, menopausal, and post-menopausal, and increased age is a risk factor for increased vulva discomfort (14). Therefore, the experiences of the participants in this study may differ from those of older female cyclists. As previously mentioned, saddle sores are seen as a taboo topic (18), which may mean the participants did not share their full experiences due to embarrassment. Additionally, we only interviewed the participants once about their experiences of saddle sores, and therefore, due to the retrospective nature, there is the possibility of recall bias (41).

The lead researcher is a female cyclist and conducted all the interviews. Her knowledge and personal experiences helped build a rapport with the participants and may have helped participants' comfort levels to share their experiences which is important in qualitative research (42). However, it is important to acknowledge that, as a co-creator of the data, the lead researcher's personal experiences had the potential to shape the direction of the interviews and exact questioning (43). The lead researcher was reflexive and discussed the findings and interpretations of the data with the research team to get an outside perspective.

### 3.9 Practical implications and recommendations

Improve education for coaches, athletes, and support staff including medical and bike fitters on female saddle sores and their prevention, treatment, and management. This would involve highlighting treatment options suggested by the participants in this study such as treatment creams, medical advice, and modifying training. Additionally, the methods participants suggested to prevent saddle sores, such as getting well-fitting cycling shorts and saddle, using anti-chafing creams, ensuring good personal hygiene, not shaving pubic hair, and regularly changing riding position.
Focus on the openness of conversations between athletes, coaches, bike fitters, and the support team, to enable female cyclists to feel more comfortable discussing saddle sores and to ensure they get advice to prevent and manage them to reduce the prevalence and severity of saddle sores for female cyclists.
More research is required on bike geometry and sizing and setup, saddle choice, cycling shorts, and chamois creams for female cyclists to reduce the incidence of saddle sores and improve female cyclists' comfort, health, and therefore enjoyment of cycling and performance.

## 4 Conclusion

Saddle sores are highly prevalent in female cyclists, and even those who said their saddle sores are manageable tolerated some pain, discomfort, swelling, and chafing. The type of saddle sores suffered by the participants was wide ranging, and most occurred in the vulva region. The causes of saddle sores were suggested to be due to pressure, friction at saddle contact points, and sweat or a combination of all three. The biggest impact of saddle sores is that they reduce the enjoyment of cycling, with 75% of the participants reporting they just put up with the discomfort and pain of saddle sores. The participants identified risk factors for developing saddle sores which included high cycling volume, ill-fitting shorts and saddle, riding in time trial position, indoor static cycling, rough road surface, wet weather conditions, and biomechanical or strength asymmetries. To treat and manage saddle sores, participants used treatment creams and modified training, and in more severe cases, they sought medical treatment or took a break from cycling. In terms of trying to prevent saddle sores, participants highlighted having well-fitting cycling shorts and saddle, using anti-chafing creams, ensuring good personal hygiene, not shaving pubic hair, and regularly changing riding position as factors that helped. The participants rode a variety of saddle shapes and often had a professional bike fit; however, many still suffered saddle discomfort. They reported the process to find a more comfortable saddle and bicycle setup as trial and error which was long and expensive. Saddle sores are thought of as a taboo topic, and the lack of female bike fitters and coaches may contribute to female riders feeling embarrassed or uncomfortable discussing exactly where is sore, meaning female riders do not seek help. Many participants had received poor or conflicting advice. These findings highlight the need for improved education on the prevention and treatment of saddle sores, and also more research into female-specific bicycle setup and saddle design.

## Data Availability

The raw data supporting the conclusions of this article will be made available by the authors, without undue reservation.
