# Supplementary material for: Female cyclists' experiences of saddle sores and their effect on cycling
Source: Front Sports Act Living. 2026 Feb 3;8:1734202. doi: 10.3389/fspor.2026.1734202 (PMC12908944; doi:10.3389/fspor.2026.1734202)
Supplement: Supplementary file 1 [file Datasheet1.docx]

# Athlete – natural cycle

Before beginning, I want to make you aware our conversation is going to be recorded, it will be kept confidential, but it helps me as I will struggle to remember everything you tell me today and will be helpful to refer back to. You can stop the recording or conversation at any point and shouldn’t feel any pressure to answer any questions, if you don’t want to answer that is absolutely no problem. There are also no right or wrong answers, I am just interested in your personal experiences relating to the menstrual cycle. We will talk through how you feel about your menstrual cycle, in relation to daily life, training and competition. This will be followed on a section on bicycle fit, injuries and saddle sores.

Do you have any questions before we start, and I will turn on the recorder?

To start a bit of background about you and cycling.

- What age are you and what cycling events do you currently compete in? Have you always been a cyclist?
- How is training and your competitions going at the moment?
- What have been your recent highlights of competing?
- What are your main goals/targets for 2024?

## Menstrual cycle

1. Thinking about when you started having your period, what was that like for you?
   1. How old were you?
   2. Were you already playing sport then?
   3. Do you remember it having any impact on your sporting participation?
   4. Was there anyone you turned to for advice or information at this time? What advice did you get?
2. As you’ve got older, has your menstrual cycle remained relatively similar and regular or have you experienced changes?
   1. If changes have happened, what has this been? (e.g., irregular period, different symptoms)
   2. Do you have any idea why?
   3. What impact have these changes had on you as an athlete?
3. Currently, what is your experience of your period like?
   1. How does it make you feel (mentally, physically, emotionally)?
   2. Do you experience any discomfort or symptoms at the different phases of your cycle? (Before, during or after menstruation?) If so, can you describe these? (e.g., cramps, headache, mood?). If it helps when I talk about symptoms these are the things I am referring too (PROVIDE LIST OF SYMPTOMS). How do you manage these symptoms?
   3. Do you consider yourself to have heavy periods? How do you find using sanitary products with training and competition? Do you ever need to use double sanitary protection? Do you have to change the product frequently? Do you feel that this impacts on your daily life, training and/or competition performance?
   4. Do you experience any changes in your core strength, coordination, mobility and flexibility throughout the menstrual cycle? Implications of this – training and competition, bicycle set-up?
4. As an athlete, how do you think about your menstrual cycle and training?
   1. Does it impact on training?
   2. How do you manage it?
   3. How have you determined how your menstrual cycle affects this? Have you/do you record your training and menstrual cycle throughout the month? Is yes, how do you complete this? Do you share this information with anyone else (do you have any privacy concerns about uses of your data by coaches and practitioners? How do you use this information?
5. How do you think about your menstrual cycle in relation to competitions?
   1. Does it impact on competitions?
   2. How do you manage it?
6. As a female athlete, have you ever had any support or advice relating to your menstrual cycle?
   1. From whom?
   2. What advice?
   3. What information would you like about menstrual cycles? What guidance or support would be useful for you as an athlete to discuss this further? From who?
7. How do you feel having conversations about your menstrual cycle?
   1. Today?
   2. With your coach, support staff, peers?
   3. Do you discuss your menstrual cycle with your coach or consider this in your training or competitions preparation? How does your coach respond?
   4. Do you feel it relevant to talk to your coach about your menstrual cycle? Why do you not discuss this with your coach? (Not relevant/haven’t thought about it/uncomfortable conversation/selection concerns?)
   5. Are you aware of elite athletes discussing the effect of their periods on their performance in the media? And if yes has this had any influence on you i.e., discussing their menstrual cycle, seeking help etc.?

## Bicycle equipment and injuries

What type of bicycle do you ride? Is it a women’s specific bike? Have you made any modifications to the bike to make it more comfortable e.g., saddle, handlebars, brake levers, stem, crank length?

Do you suffer from any cycling overuse injuries, e.g., lower back pain, knee pain, saddle sores, foot pain, hand/finger numbness? Are there any types of training and racing which make these injuries worse? Have you modified your bicycle position to address these?

What type of saddle do you use? Do you have different saddles depending on your position (e.g., road racing, TT, track)?

Have you suffered from saddle sores? Where have you had saddle sores e.g., sit bones, perineum, labia, vulva, upper inner thigh (show diagram below to aid discussion)? What type of saddle sores e.g., chafing, pressure/bruising, swelling of labia, infected skin/hair follicles, cyst like lumps and boils, ulcerations, nodules, abscess, numbness?

How have you treated your saddle sores e.g., repair gel, time off the bike, painkillers, antibiotics, medical treatment (draining abscess), surgery?

Are there any factors that increase the likelihood of you developing a saddle sore?

Have saddle sores affected your ability to train and compete? Influence your enjoyment of cycling?

Do you discuss any saddle sore issues with your coach?

Have you done anything to prevent saddle sores e.g., change saddle/saddle position, bicycle set-up, type of cycling shorts (chamois choice), chammie cream, avoid waxing pubic hair, physiotherapy if leg imbalance/pelvic twist?

# Final

1. Are then any areas you would like researching in regard to female cycling e.g., menstrual cycle, training, bicycle set-up, saddle choice, injuries?

I am just going to double check we have covered everything today.

Thank you, those are all my questions for now, but is there anything else you would like to share or add about your experience of menstrual cycle, training and performance?

The conversation is now finished, and I will stop recording. Thank you for taking the time I really appreciate it.

# Athlete – contraceptives

Before beginning, I want to make you aware our conversation is going to be recorded, it will be kept confidential, but it helps me as I will struggle to remember everything you tell me today and will be helpful to refer back to. You can stop the recording or conversation at any point and shouldn’t feel any pressure to answer any questions, if you don’t want to answer that is absolutely no problem. There are also no right or wrong answers, I am just interested in your personal experiences relating to the menstrual cycle. We will talk through how you feel about your menstrual cycle, in relation to daily life, training and competition. This will be followed on a section on bicycle fit, injuries and saddle sores

Do you have any questions before we start, and I will turn on the recorder?

To start a bit of background about you and cycling.

- What age are you and what cycling events do you currently compete in? Have you always been a cyclist?
- How is training and your competitions going at the moment?
- What have been your recent highlights of competing?
- What are your main goals/targets for 2024?

1. Thinking about when you started having your period, what was that like for you?
   1. How old were you?
   2. Were you already playing sport then?
   3. Do you remember it having any impact on your sporting participation?
   4. Was there anyone you turned to for advice or information at this time? What advice did you get?
   5. Did you experience any symptoms or discomfort (cramps, headache, change in mood)?
   6. Did you consider yourself to have heavy periods? How did you find using sanitary products with training and competition? Did this impact daily life, training or competition?
2. You have shared that you take contraceptives.
   1. Do you experience withdraw bleeds and are these regular? Does any irregularity affect daily life, training or competition?
   2. What was your reasoning for taking contraceptives, was this related to sport (e.g., to manage symptoms, control timing for sport or purely for contraceptive reasons)?
   3. What impacted your decision as to which form of contraception to take? Did you receive any advice or information about this? Discuss it with anyone?
   4. Do you experience any symptoms, if it helps when I talk about symptoms these are the things I am referring too (PROVIDE LIST OF SYMPTOMS) How did you manage these symptoms?
3. As an athlete, what are your thoughts about having a period during training?
4. As an athlete, what are your thoughts about having a period during competition?
   1. How do you manage your withdraw bleeds at competition? Does it affect your performance?
5. As a female athlete, have you ever had any support or advice relating to your menstrual cycle?
   1. From whom?
   2. What advice?
   3. What information would you like about menstrual cycles? What guidance or support would be useful for you as an athlete to discuss this further? Who from?
6. How do you feel having conversations about your menstrual cycle?
   1. Today?
   2. With your coach, support staff, peers?
   3. Do you discuss your menstrual cycle with your coach, regarding taking contraceptives? Do you consider how taking contraceptives may interact with training and performance?
   4. Do you feel it relevant to talk to your coach about your menstrual cycle? If yes, why. If no, why?
   5. Are you aware of elite athletes discussing the effect of their periods on their performance in the media? And if yes has this had any influence on you i.e., discussing their menstrual cycle, seeking help etc.?

## Bicycle equipment and injuries

What type of bicycle do you ride? Is it a women’s specific bike? Have you made any modifications to the bike to make it more comfortable e.g., saddle, handlebars, brake levers, stem, crank length?

Do you suffer from any cycling overuse injuries, e.g., lower back pain, knee pain, saddle sores, foot pain, hand/finger numbness? Are there any types of training and racing which make these injuries worse? Have you modified your bicycle position to address these?

What type of saddle do you use? Do you have different saddles depending on your position (e.g., road racing, TT, track)?

Have you suffered from saddle sores? Where have you had saddle sores e.g., sit bones, perineum, labia, vulva, upper inner thigh (show diagram below to aid discussion)? What type of saddle sores e.g., chafing, pressure/bruising, swelling of labia, infected skin/hair follicles, cyst like lumps and boils, ulcerations, nodules, abscess, numbness?

How have you treated your saddle sores e.g., repair gel, time off the bike, painkillers, antibiotics, medical treatment (draining abscess), surgery?

Are there any factors that increase the likelihood of you developing a saddle sore?

Have saddle sores affected your ability to train and compete? Influence your enjoyment of cycling?

Do you discuss any saddle sore issues with your coach?

Have you done anything to prevent saddle sores e.g., change saddle/saddle position, bicycle set-up, type of cycling shorts (chamois choice), chammie cream, avoid waxing pubic hair, physiotherapy if leg imbalance/pelvic twist?

# Final

1. Are then any areas you would like researching in regard to female cycling e.g., menstrual cycle, training, bicycle set-up, saddle choice, injuries?

I am just going to double check we have covered everything today.

Thank you, those are all my questions for now, but is there anything else you would like to share or add about your experience of menstrual cycle, training, and performance?

The conversation is now finished, and I will stop recording. Thank you for taking the time I really appreciate it.

# Symptom list:

Pelvic or abdominal cramp

Back pain

Breast pain/tenderness

Joint pain/ muscle aches and cramps

Discomfort

Flooding

Heavy bleeding

Headaches/migraines

Dizziness/ light headiness / reduced coordination

Clumsiness

Tiredness/ fatigue / decreased energy levels

Bloating

Nausea (feeling sick)

Constipation

Diarrhoea

Water retention

Weight gain

Sleep disturbance

Temperature fluctuations

Changes to /difficulties breathing

Worry

Anxiety

Distraction

Poor concentration/ memory

Emotional

Moody

Agitated / Irritable / Restlessness

Reduced motivation

Cravings / changes in appetite
